# Comparison of clinical outcomes of drug-coated balloons angioplasty vs. plain old balloons angioplasty for peripheral arterial disease: an umbrella meta-analysis

**DOI:** 10.3389/fcvm.2024.1511268

**Published:** 2024-11-21

**Authors:** Jiacheng Li, Wei Lu, Lihong Lin, Jiawen Wu, Guobing Cheng, Qiang Hu, Yi Guo

**Affiliations:** ^1^ Department of Vascular Surgery, The Quzhou Affiliated Hospital of Wenzhou Medical University, Quzhou People’s Hospital, Quzhou, Zhejiang, China; ^2^ Department of Nosocomial Infection Control, The Quzhou Affiliated Hospital of Wenzhou Medical University, Quzhou People’s Hospital, Quzhou, Zhejiang, China

**Keywords:** balloon angioplasty, paclitaxel, peripheral arterial disease, systematic review, umbrella review

## Abstract

**Background:**

Peripheral artery disease (PAD) affects millions globally, causing significant morbidity. Traditional treatments like plain old balloon angioplasty (POBA) have limited success due to high restenosis rates. Drug-coated balloon angioplasty (DCBA) has emerged as a promising alternative, locally delivering antiproliferative drugs like paclitaxel to reduce restenosis. However, the clinical outcomes of DCBA compared to POBA remain inconsistent across various studies.

**Objective:**

This umbrella meta-analysis aimed to compare the clinical outcomes of DCBA and POBA in PAD patients, synthesizing data from multiple meta-analyses to provide a more robust evidence base.

**Methods:**

We conducted an umbrella meta-analysis following PRISMA guidelines, systematically reviewing Cochrane Library, Embase, PubMed, and Web of Science. Studies were included if they compared DCBA and POBA in PAD patients, focusing on primary outcomes such as target lesion revascularization (TLR), primary patency (PP), all-cause mortality (ACM), and amputation. Secondary outcomes included restenosis, late lumen loss (LLL), and major adverse events (MAE).

**Results:**

Sixteen meta-analyses were included. DCBA significantly reduced the risk of TLR (OR: 0.41, 95% CI: 0.34–0.49), PP was significantly higher in DCBA (OR: 2.05, 95% CI: 1.53–2.75), and restenosis was lower (OR: 0.46, 95% CI: 0.41–0.51). No significant differences were found in ACM or amputation risk between the two groups. Heterogeneity was moderate to high across most outcomes.

**Conclusion:**

DCBA provides significant advantages over POBA in reducing TLR and restenosis while maintaining vessel patency. However, the effects on ACM and amputation remain inconclusive. Future research should focus on long-term safety and identifying which patient subgroups benefit most from DCBA.

**Systematic Review Registration:**

https://www.crd.york.ac.uk/, PROSPERO [CRD42024591967].

## Background

Peripheral artery disease (PAD) is a significant manifestation of atherosclerosis, affecting over 202 million people worldwide, with a particularly higher disease burden in low-income and middle-income countries (1, 2). It not only leads to intermittent claudication but also significantly increases the risk of heart disease and stroke, thereby affecting patients’ quality of life (3).

Conventional treatments include pharmacotherapy and percutaneous transluminal angioplasty (PTA), with plain old balloon angioplasty (POBA) being a commonly used interventional therapy. However, the effectiveness of POBA in restoring blood flow is limited by restenosis and vasoconstriction, with restenosis rates reaching as high as 30% to 60% (4). With the introduction of new technologies such as drug-coated balloon angioplasty (DCBA), clinical reliance on POBA has gradually diminished. This treatment works by locally releasing antiproliferative drugs, particularly paclitaxel, to inhibit neointimal hyperplasia, thereby reducing the risk of restenosis. This mechanism allows DCBA to demonstrate potential advantages in the treatment of PAD, especially in complex lesions and high-risk patients (5, 6).

Recently, several systematic reviews and meta-analyses have evaluated the efficacy of DCBA compared to POBA in the treatment of PAD, but the conclusions of these studies have not been entirely consistent. In 2022, Cai et al. (7) evaluated the efficacy of DCBA compared to POBA and found that DCBA did not reduce the risk of target lesion revascularization (TLR) (*OR*: 0.72, 95% *CI*: 0.35–1.45), or amputation (*OR*: 1.34, 95% *CI*: 0.64–2.79). However, in 2019, Caradu et al. (8) reported that DCBA significantly reduced the risk of TLR (*OR*: 0.29, 95% *CI*: 0.20–0.40). And in 2022, Ullah et al. (9) concluded that DCBA also significantly lowered the risk of amputation (*OR*: 0.68, 95% *CI*: 0.47–0.99). Additionally, there were inconsistent conclusions regarding key outcomes such as primary patency (PP) (8, 10), and all-cause mortality (ACM) (11, 12), as well as secondary outcomes including major adverse events (MAE) (13, 14), restenosis (15, 16), late lumen loss (LLL) (10, 12) and ankle-brachial index (ABI) (9, 12).

Umbrella meta-analysis aims to synthesize the results of multiple related meta-analyses, providing a more comprehensive evidence base (17, 18). When conclusions across meta-analyses are inconsistent, an umbrella meta-analysis becomes particularly necessary, as it can uncover heterogeneity and potential biases between studies, enhancing the reliability and generalizability of conclusions. This approach not only evaluates the efficacy of interventions but also considers methodological quality, helping researchers to better understand the clinical effects of treatment strategies (19).

Given the current controversies in research, the objectives of this study are as follows: First, to comprehensively assess the evidence on the use of DCBA vs. POBA in PAD. Second, for the first time, to systematically compare the clinical outcomes of DCBA and POBA through an umbrella meta-analysis, revealing the advantages or limitations of DCBA and POBA in PAD. We will synthesize existing meta-analysis results to clarify the efficacy of DCBA in primary outcomes such as TLR, PP, ACM, and amputation, and analyze the heterogeneity and potential biases across different studies. The goal is to provide more reliable evidence for clinical practice.

## Materials and methods

### Study registration

The aim of the umbrella meta-analysis was to provide a broad comparison of the clinical outcomes between DCBA and POBA in PAD. It was undertaken according to PRISMA criteria (18). Please see the checklist in Supplementary Table S1. This study had been registered in PROSPERO with number CRD42024591967.

### Search strategy

We searched Cochrane Library, Embase, PubMed, and Web of Science without language limitations, using the terms “peripheral arterial disease” AND “drug coated ballon” AND “meta analy*”, along with manual retrieval. The time frame of the search was January 1, 2014 to October 1, 2024. Please see the search strategy in Supplementary Table S2.

### Inclusion and exclusion criteria

All potentially eligible studies were examined. The following criteria were established to select relevant articles.

#### Inclusion criteria

(a) patients presenting with symptoms of PAD (intermittent claudication or critical limb ischemia documented by digital subtraction angiography); (b) only drug coated ballon angioplasty was considered as intervention and plain old balloon angioplasty was considered as control; (d) meta-analyses enrolling RCTs or cohort studies; (e) the pooled effect size was *OR* or *RR*, and *MD* or *SMD*.

#### Exclusion criteria

(a) original articles; (b) net-work meta-analysis; (c) unusable information; (d) meta-analyses with low quality.

Two researchers (JL and QH) individually screened titles and abstracts, and then read full texts of relevant publications for eligibility. Any disagreement in literature selection was resolved by consulting the senior investigator (YG).

### Quality assessment

A MeaSurement Tool to Assess systematic Review 2 (AMSTAR 2) was used to evaluate the quality of included meta-analyses (20). It is a 16-item tool used to evaluate the methodological quality of systematic reviews, especially those involving RCTs. It assesses key aspects such as protocol registration, search strategy, bias risk, and statistical methods. Each item was given a score of 1 if the specific criterion was met or partially met, and 0 if the criterion was not met or information was unclear and the total score was categorized as high quality (13–16), moderate (9–12), low (5–8), or critically low (0–4) (21). The assessment was done by WL and LL independently. Any disagreement was resolved by YG.

### Data extraction

Two researchers (JW and GC) independently extracted the following information using a pre-made data collection sheet: first, the characteristics in each meta-analysis, such as author, title, publication year, participants, intervention, control, outcome, study type included; second, characteristics of each outcome, such as sample size, effect size and corresponding 95% confidence interval (*CI*) of each outcome. Definitions of primary and secondary outcomes were shown in Supplementary Table S3.

### Data analysis

Meta-analysis was conducted using the meta and metafor packages of R Project Version 4.4.1. The pooled effect size for dichotomous outcomes was represented by odds ratio (*OR*) and its 95% *CI*. Due to the low incidence of outcomes reported in the included studies, relative risk (*RR*) was approximately treated as *OR* for the purpose of the meta-analysis. The pooled effect size for continuous outcomes was represented by the standardized mean difference (*SMD*) and its 95% *CI*. The DerSimonian-Laird random effect model was used for various designs across meta-analyses (22). Heterogeneity was tested by Cochran's *Q*-test and *I*^2^: the analysis was considered as low heterogeneity if *P* ≤ 0.1 and *I*^2^ > 50%, otherwise it was considered as high heterogeneity. Sensitivity analysis was done by removing each meta-analysis separately. Publication bias was tested by the funnel plot and Egger linear regression test for analyses with more than 10 meta-analyses included. Finally, subgroup analysis was used to compare the differences in primary outcomes and explore the potential heterogeneity according to length of follow-up and paclitaxel dose of DCBA.

## Results

### Study characteristics

**Figure 1:**
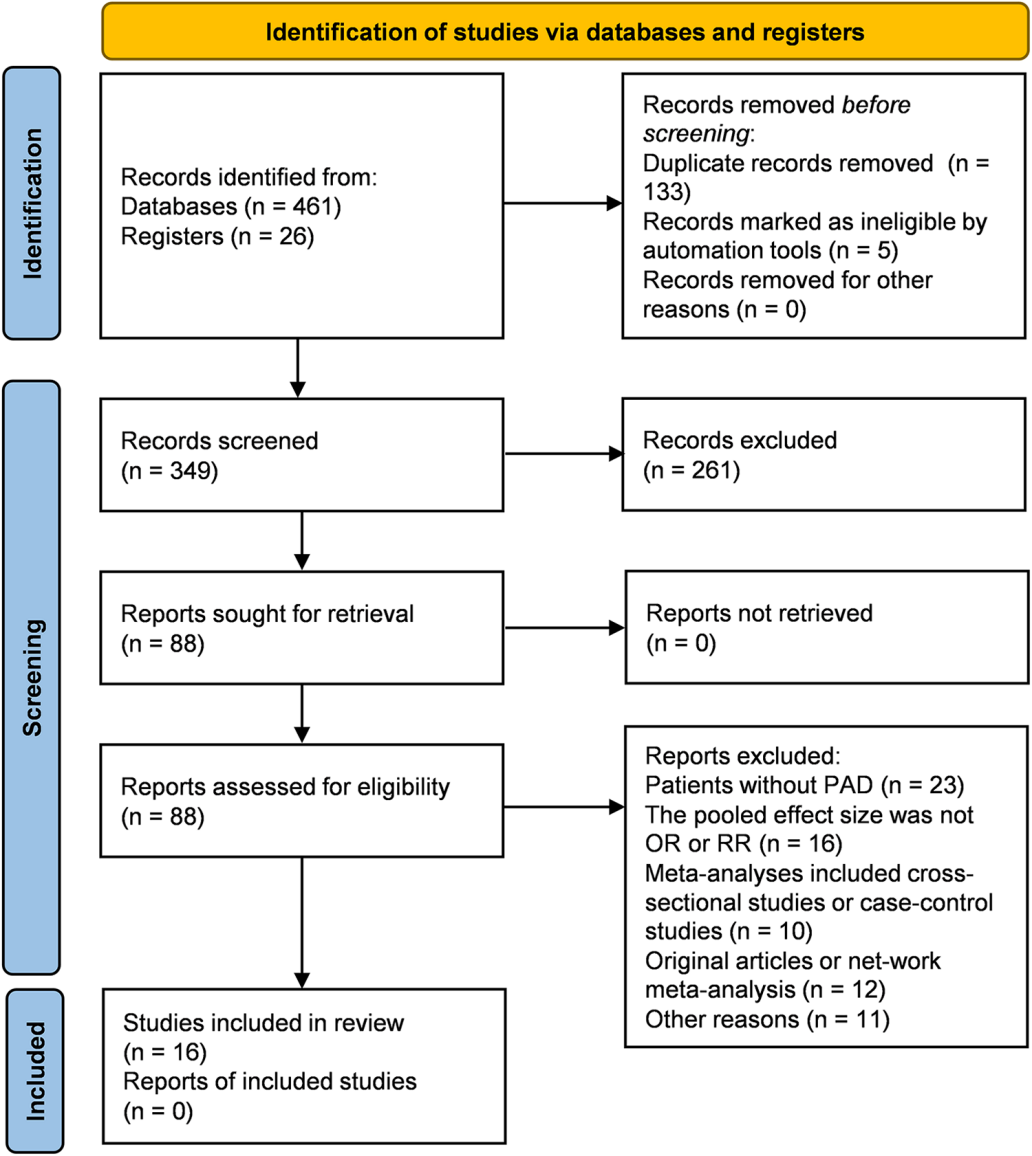
Study selection flowchart.

16 unique studies between 2016 and 2024 were eligible for this umbrella meta-analysis. Please see the study selection flowchart in Figure 1. The characteristics of meta-analyses were shown in Table 1. 4 studies included both RCT and cohort study. 13, 6, 13, 10 meta-analyses provided data regarding TLR, PP, ACM, and amputation separately. Meanwhile, 3, 9, 4, 3 meta-analyses provided data regarding MAE, restenosis, LLL and ABI separately. The AMSTAR 2 scores of the included studies included from 8 to 15, with most studies being of moderate to high quality. Quality of studies was shown in Supplementary Table S4.

**Table 1:** Characteristics of included meta-analyses.

Author, year	Study type	Outcomes	Effect sizes	Number of included studies/sample size	*I*^2^ (%)	Effect size (95% *CI*)	AMSTAR 2 score
Katsanos et al. (23)	RCT	TLR	RR	11/1,775	61.0	0.33 (0.22, 0.49)	15
		Restenosis	RR	10/1,422	47.0	0.47 (0.37, 0.61)	
		Amputation	RR	10/1,566	0.0	0.99 (0.27, 3.53)	
		LLL	MD	8/563	62.0	−0.89 (−1.14, −0.64)	
Cassese et al. (13)	RCT	TLR	RR	5/609	39.0	0.71 (0.47, 1.09)	9
		ACM	RR	5/612	0.0	1.14 (0.71, 1.82)	
		MAE	RR	4/562	67.0	0.92 (0.59, 1.43)	
		Amputation	RR	5/614	0.0	1.04 (0.70, 1.54)	
Caradu et al. (8)	RCT, cohort study	TLR	OR	8/1,368	29.0	0.29 (0.20, 0.40)	12
		PP	OR	7/1,281	44.0	0.38 (0.27, 0.54)	
		Restenosis	OR	9/1,501	36.0	0.36 (0.26, 0.51)	
Klumb et al. (12)	RCT	PP	RR	5/974	45.0	1.49 (1.26, 1.77)	13
		ACM	RR	7/1,160	0.0	1.53 (0.94, 2.50)	
Klumb et al. (12)	RCT	Amputation	RR	5/349	0.0	2.32 (0.69, 7.85)	13
		LLL	MD	8/709	78.0	−0.97 (−1.33, −0.61)	
		ABI	MD	7/1,454	49.0	0.01 (−0.03, 0.05)	
Varetto et al. (24)	RCT, cohort study	TLR	OR	12/2,191	58.0	0.28 (0.18, 0.43)	12
		PP	OR	12/1,967	63.0	3.17 (2.10, 4.76)	
Anantha-Narayanan et al. (25)	RCT	TLR	RR	22/3,127	62.0	0.49 (0.40, 0.61)	13
		ACM	RR	17/2,849	0.0	1.33 (0.97, 1.84)	
		Restenosis	RR	15/2,108	64.0	0.50 (0.39, 0.64)	
		Amputation	RR	8/1,050	0.0	0.75 (0.28, 2.02)	
		LLL	MD	12/1,276	55.0	−0.87 (−1.05, −0.69)	
Ipema et al. (15)	RCT, cohort study	TLR	OR	4/625	80.47	0.36 (0.12, 1.12)	11
		ACM	OR	5/686	0.0	1.46 (0.83, 2.55)	
		Restenosis	OR	4/398	86.9	0.35 (0.10, 1.21)	
		Amputation	OR	5/680	0.1	1.08 (0.45, 2.60)	
Dinh et al. (26)	RCT	ACM	RR	34/7,654	0.0	1.07 (0.96, 1.20)	14
Cao et al. (27)	RCT	TLR	RR	6/470	0.0	0.38 (0.27, 0.54)	14
		ACM	RR	5/424	0.0	1.12 (0.51, 2.48)	
		Restenosis	RR	4/297	7.0	0.45 (0.33, 0.63)	
		Amputation	RR	5/439	0.0	0.90 (0.16, 5.18)	
Zhang and Yin (28)	RCT	ACM	RR	8/2,165	28.0	1.20 (0.98, 1.48)	13
Cai et al. (7)	RCT	TLR	OR	3/514	41.3	0.72 (0.35, 1.45)	14
		ACM	OR	4/650	0.0	1.30 (0.69, 2.46)	
		MAE	OR	4/653	64.6	0.68 (0.36, 1.31)	
		Amputation	OR	4/649	0.0	1.34 (0.64, 2.79)	
Barbarawi et al. (14)	RCT	TLR	RR	8/1,391	68.0	0.54 (0.35, 0.84)	11
		PP	RR	3/364	82.0	2.12 (1.01, 4.42)	
		ACM	RR	9/1,490	36.0	1.11 (0.73, 1.69)	
		MAE	RR	7/1,279	64.0	0.69 (0.48, 1.00)	
Barbarawi et al. (14)	RCT	Restenosis	RR	7/943	70.0	0.53 (0.37, 0.76)	11
		Amputation	RR	9/1,523	0.0	1.32 (0.85, 2.08)	
Ullah et al. (9)	RCT, cohort study	TLR	OR	35/4,133	21.7	0.38 (0.31, 0.47)	8
		ACM	OR	37/11,063	0.0	0.96 (0.85, 1.09)	
		Restenosis	OR	22/2,001	25.3	0.46 (0.37, 0.57)	
		Amputation	OR	30/3,579	0.0	0.68 (0.47, 0.99)	
		ABI	MD	12/1,486	98.5	0.37 (−0.53, 1.28)	
Zhen et al. (16)	RCT	TLR	OR	4/302	39.0	0.21 (0.09, 0.49)	10
		ACM	OR	4/315	0.0	1.07 (0.31, 3.12)	
		Restenosis	OR	3/204	0.0	0.31 (0.16, 0.61)	
		ABI	MD	3/202	79.0	0.02 (−0.11, 0.14)	
Koeckerling et al. (11)	RCT	TLR	OR	10/1,578	41.7	0.42 (0.29, 0.60)	13
		PP	OR	10/1,614	2.6	2.47 (1.93, 3.16)	
		ACM	OR	12/1,894	0.0	0.96 (0.67, 1.39)	
Cui and Wu (10)	RCT	TLR	RR	7/930	56.0	0.61 (0.38, 0.96)	12
		PP	RR	4/699	73.0	1.32 (1.01, 1.71)	
		ACM	RR	8/1,420	0.0	1.14 (0.82, 1.59)	
		Restenosis	RR	3/284	87.0	0.70 (0.35, 1.37)	
		Amputation	RR	7/959	0.0	1.62 (0.94, 2.78)	

Abbreviations: ABI, ankle-brachial index; ACM, all-cause mortality; LLL, late lumen loss; MAE, major adverse event; MD, mean difference; OR, odds ratio; PP, primary patency; RCT, randomized controlled trail; RR, risk ratio; TLR, target lesion revascularization.

### Main analysis of clinical outcomes

**Figure 2:**
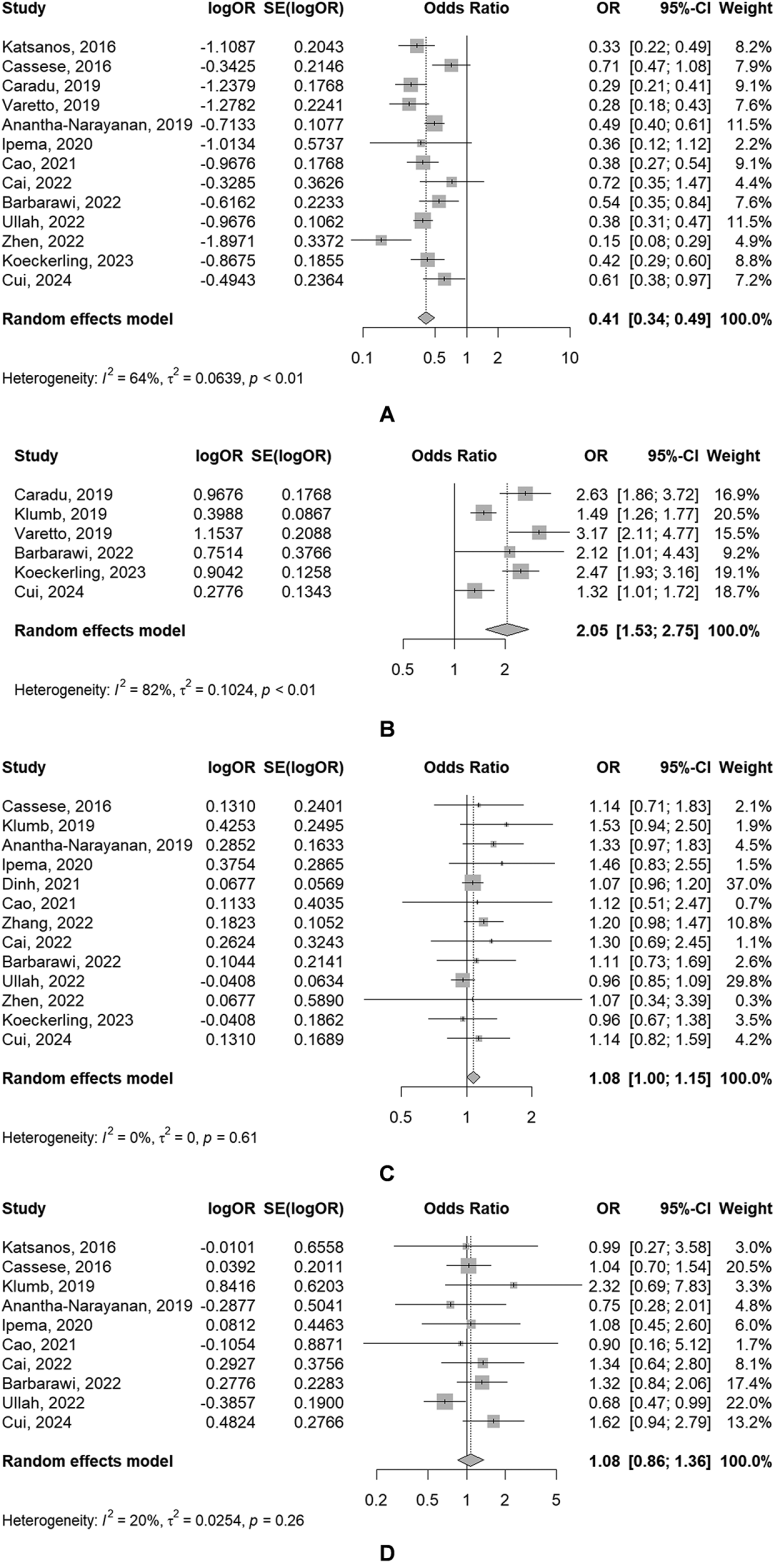
Forest plots of meta-analyses of the primary outcomes [**(A)** TLR; **(B)** PP; **(C)** AMC; **(D)** amputation].

**Figure 3:**
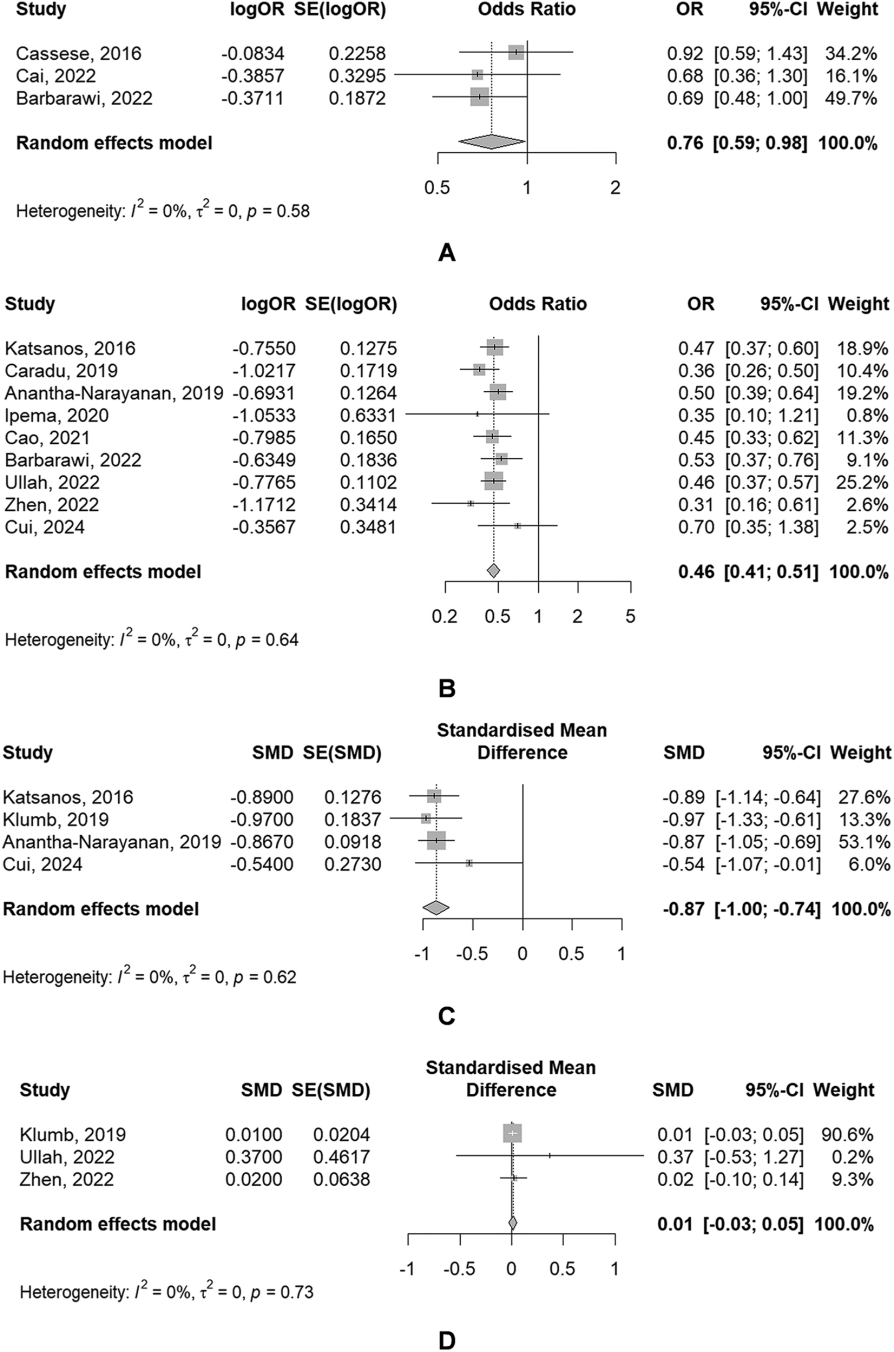
Forest plots of meta-analyses of secondary outcomes [**(A)** MAE; **(B)** restenosis; **(C)** LLL; **(D)** ABI].

The main analysis of primary outcome, secondary outcomes between DCBA and POBA was shown in Figures 2, 3 separately. All main analysis results were summarized in Supplementary Table S5.

#### Primary outcomes

(1) TLR

The use of DBC was associated with a significantly reduced risk of TLR (*OR*: 0.41, 95% *CI*: 0.34–0.49), of which showed a significant heterogeneity (*P* = 0.008, *I*^2^ = 64.3%) (Figure 2A).

(2) PP

PP rate in DCBA groups was significantly higher than that in POBA groups (*OR*: 2.05, 95% *CI*: 1.53–2.75), of which also showed a significant heterogeneity (*P* < 0.001, *I*^2^ = 82.5%) (Figure 2B).

(3) ACM

The use of DBC was associated with a slightly increased risk of ACM (*OR*: 1.08, 95% *CI*: 1.00–1.15), but the result was not statistically significant. And significant heterogeneity was absent (*P* = 0.614, *I*^2^ = 0.0%) (Figure 2C).

(4) Amputation

There was no significant difference of the risk of amputation between DCBA and POBA (*OR*: 1.08, 95% *CI*: 0.86–1.36), with the absence of significant heterogeneity (*P* = 0.260, *I*^2^ = 19.9%) (Figure 2D).

#### Secondary outcomes

(1) MAE

The use of DBC was associated with a significantly reduced risk of MAE (*OR*: 0.76, 95% *CI*: 0.59–0.98), of which showed a significant heterogeneity (*P* = 0.578, *I*^2^ = 0.0%) (Figure 3A).

(2) Restenosis

The use of DBC was associated with a significantly reduced risk of restenosis (*OR*: 0.46, 95% *CI*: 0.41–0.51), with the absence of significant heterogeneity (*P* = 0.637, *I*^2^ = 0.0%) (Figure 3B).

(3) LLL

4 The use of DBC was associated with a significantly lower LLL (*SMD*: −0.87, 95% *CI*: −1.00 to −0.74), with the absence of significant heterogeneity (*P* = 0.619, *I*^2^ = 0.0%) (Figure 3C).

(4) ABI

3 meta-analyses provided data regarding ABI. There was no significant difference of ABI between DCBA and POBA (*SMD*: 0.01, 95% *CI*: −0.03 to 0.05), with the absence of significant heterogeneity (*P* = 0.731, *I*^2^ = 0.0%) (Figure 3D).

### Sensitivity analysis

Results of sensitivity analysis of meta-analyses of primary outcomes and secondary outcomes were shown in Supplementary Figures S1, S2 separately.

There were no significantly changes in the pooled estimates of primary outcomes when omitting included meta-analysis individually, which indicated that the analyses of primary outcomes were quite robust. However, in the analysis of amputation, when omitting Ullah, 2022, the heterogeneity decreased, which indicated that it might be a source of heterogeneity.

Similar to the results of primary outcomes, there were no significantly changed in the pooled estimates of secondary outcomes, either, which indicated that the analyses of secondary outcomes were quite robust.

### Publication bias

The publication bias was tested in TLR, ACM, and amputation, of which the funnel plots were shown in Supplementary Figure S3. Egger linear regression test showed that there existed the publication bias in meta-analyses of ACM (*t* = 2.20, *P* = 0.050). There was no publication bias in meta-analysis of TLR (*t* = −0.29, *P* = 0.780) and amputation (*t* = 0.81, *P* = 0.441).

### Subgroup analysis of primary outcomes

The results of the subgroup analyses were shown in Table 2. For TLR, when grouped by length of follow-up, heterogeneity decreased compared to the main analysis, with no significant differences in risk among the groups. When stratified by paclitaxel dosage, the 3.0–3.5 μg/mm^2^ group also showed decreased heterogeneity, but only this group significantly reduced the risk of TLR.

**Table 2:** Results of subgroup analyses of primary outcomes.

Outcomes	Subgroups	Number of included meta-analyses	*I*^2^ (%)	*P*	Pooled *OR* (95% *CI*)
TLR					
Length of follow-up (month)	6	6	47.3	0.091	0.40 (0.30, 0.53)
	12	9	57.4	0.016	0.35 (0.28, 0.45)
	24	3	44.3	0.166	0.34 (0.27, 0.43)
Paclitaxel dose (μg/mm^2^)	2.0	2	75.4	0.044	0.51 (0.23, 1.14)
	3.0–3.5	2	42.7	0.187	0.23 (0.16, 0.32)
PP					
Length of follow-up (month)	12	5	89.0	<0.001	2.34 (1.54, 3.56)
	24	3	89.9	<0.001	2.18 (1.39, 3.41)
ACM					
Length of follow-up (month)	12	9	0.0	0.937	1.03 (0.92, 1.15)
	24	5	6.5	0.370	1.07 (0.96, 1.19)
	60	3	83.6	0.002	1.06 (0.84, 1.34)
Paclitaxel dose (μg/mm^2^)	2.0	3	0.0	0.794	1.09 (1.04, 1.15)
	3.0	3	73.1	0.024	1.15 (0.83, 1.60)
	3.5	3	56.0	0.103	1.40 (0.99, 1.97)
Amputation					
Length of follow-up (month)	12	5	8.7	0.357	1.24 (0.86, 1.80)
	24	2	71.0	0.063	1.09 (0.32, 3.72)

Regarding PP, there was no significant change in heterogeneity when grouped by length of follow-up, and no notable differences in risk between the two groups.

For ACM, when grouped by length of follow-up, all three groups indicated that the use of DCBA was not associated with an increased risk of mortality. When stratified by paclitaxel dosage, there were no significant differences in mortality risk between the three groups, but only the 2.0 μg/mm^2^ group would increase risk of ACM.

For amputation, when analyzed by length of follow-up, there were also no significant differences in amputation risk between the two groups.

## Discussion

This study, through the first umbrella meta-analysis, offered a comprehensive comparison of clinical outcomes between DCBA and POBA, providing a solid evidence base for clinical decision-making. The main analysis showed that the use of DCBA could significantly reduce the risk of TLR, MAE, restenosis and LLL and improve PP in the same time. These advantages were mainly attributed to the localized antiproliferative effect of paclitaxel. Compared to POBA, DCBA maintained its therapeutic efficacy over specific follow-up periods, with higher doses of paclitaxel (3.0–3.5 μg/mm^2^) notably enhancing this antiproliferative effect. This study provides robust evidence for clinical practice, supporting the use of DCBA in PAD treatment, particularly for patients at high risk of restenosis.

This study's main analysis revealed that compared to POBA, DCBA significantly reduced the risk of TLR, MAE, restenosis, and LLL, while also demonstrating a marked increase in PP. These effects are primarily attributed to the localized release of paclitaxel, an antiproliferative drug that effectively inhibited the proliferation of smooth muscle cells, thereby reducing the risk of neointimal hyperplasia (13), which further maintains vessel patency. Moreover, the specialized coating carrier in DCBA ensures that paclitaxel is efficiently transferred to the vessel wall during balloon expansion, minimizing drug loss and increasing drug concentration at the treatment site (29). Even after the angioplasty procedure, paclitaxel continues to suppress neointimal hyperplasia, preventing restenosis (30). Subgroup analysis based on paclitaxel dosage revealed that higher doses (3.0–3.5 μg/mm^2^) further enhanced the efficacy of DCBA, providing a stronger antiproliferative effect and greater patency maintenance than lower doses. This suggests that adequate drug concentration at the lesion site is crucial for maximizing DCBA's clinical benefits. The findings of this study were, to some extent, consistent with previous research (29–31), which also demonstrated that DCBA maintained its inhibitory effect on TLR, LLL, and facilitation on PP over extended periods, further supporting the sustained efficacy of DCBA. Although the three included studies showed no statistically significant difference between DCBA and POBA in terms of MAE, it is noteworthy that Barbarawi et al. (14) reported an upper confidence interval limit of 1, indicating the possibility of a false-negative result due to insufficient statistical power (32). Therefore, this study utilized an umbrella meta-analysis to synthesize more research findings, increase the sample size, and enhance the statistical power, thereby providing a clearer assessment of the effect of DCBA in reducing MAE.

However, DCBA didn't show significant advantages in reducing the risk of ACM, amputation and increasing ABI. ACM risk of DCBA was slightly higher than of POBA, though no statistically significant differences were observed between the two groups. Although DCBA performs well in local treatment, long-term use may have unforeseen systemic effects, such as systemic drug reactions and potential side effects (31). Additionally, PAD patients typically have comorbid cardiovascular diseases, which may also affect their post-treatment survival rates. These factors suggest that clinicians should carefully consider the overall health status of patients when using DCBA and continuously monitor and assess treatment outcomes and potential mortality risks (4). And point estimates of amputation suggested a slightly higher risk in DCBA compared to that in POBA. This could be related to systemic reactions caused by drug release (31). Such potential risks may be more pronounced in PAD patients with other comorbidities, particularly those with diabetes (33) or chronic kidney disease (34). In addition, there was considerable heterogeneity across included meta-analyses on ABI. For example, Ullah et al. (9) reported an *I*^2^ of 98% for ABI, making it difficult to draw unified conclusions. High heterogeneity suggests significant differences in patient characteristics, treatment methods, and length of follow-up across studies, potentially influencing results. We utilized an umbrella meta-analysis to synthesize a broader range of studies, clarifying the true impact of both treatment methods on ABI. This approach helped overcome the uncertainties that might arise from high heterogeneity in individual meta-analyses, thereby providing more convincing evidence. ABI is an indicator of lower limb vascular patency, reflecting changes in vascular resistance (35). Both DCB and POBA improve hemodynamics immediately through the mechanical expansion of the balloon. Thus, during early follow-up, there is typically no significant difference in ABI improvement between the two treatments. Furthermore, ABI may not be sensitive enough to detect subtle changes in vascular patency in some patients, particularly in the short term (36). As a result, even though DCB is more effective at preventing restenosis, these advantages may not be immediately reflected in ABI changes.

While DCBA demonstrated significant benefits in improvement on vessel patency, potential long-term risks associated with paclitaxel-coated devices need careful consideration. Although the analysis found no statistically significant differences between two groups in ACM, or amputation, certain risk factors associated with paclitaxel may necessitate cautious clinical application, particularly over extended periods. Systemic absorption of paclitaxel poses a potential risk for systemic toxicity, particularly at lower doses that might not maintain effective local concentrations yet still carry the potential for adverse reactions (37). For example, subgroup analysis indicated that lower doses could lead to incomplete antiproliferative effects at the lesion site, while also raising the risk of mild systemic toxicity. This includes potential endothelial damage, inflammatory responses, or increased thrombosis risk (38), which could elevate ACM for patients with coexisting cardiovascular conditions. In addition, the point estimates of amputation suggested a slight increase in the risk of amputation associated with DCBA. This may be attributable to systemic reactions stemming from paclitaxel's release, which might affect patients with diabetes or other chronic conditions more acutely (39).

This study has several limitations: First, the quality of the included studies was assessed using AMSTAR 2, and a small number of studies were of moderate quality, which may have affected the overall reliability of the conclusions to some extent. Second, due to the limitations of data types, we were unable to perform subgroup analyses based on patient-related characteristics (such as lesion location, lesion length, and comorbidities) to evaluate outcome differences in patients with varying characteristics and explore potential sources of heterogeneity. Third, the study primarily focused on comparing DCBA and POBA, but in clinical practice, many PAD patients may undergo multiple interventions (such as stent implantation or pharmacotherapy). Since we were unable to fully account for these combined interventions, the generalizability of the study results may be somewhat limited. Sirolimus-coated balloons angioplasty (SCBA) have demonstrated much promise as an alternative drug eluting device to existing paclitaxel coated balloon angioplasty (PCBA) for the treatment of PAD (40). However, the existing meta-analyses only compared the effect of PCBA with POBA. Due to the limitation of data availability, the study didn't consider the equivalence of SCBA and PCBA. Lastly, the study only searched four databases, which may have introduced publication bias to some extent.

## Conclusions

This study is the first to compare the clinical outcomes of DCBA and POBA through an umbrella meta-analysis. The results demonstrated that DCBA significantly reduced the risk of TLR, restenosis, and MAE, decreased LLL, and improved PP, while no significant differences were found between the two groups in terms of ACM, amputation, or ABI. Additionally, the therapeutic efficacy of DCBA was influenced by length of follow-up and paclitaxel dosage. Future research should further investigate the efficacy of DCBA in different PAD patient populations, examining the impact of factors such as lesion location, lesion length, patient age, gender, and comorbidities (e.g., diabetes and renal insufficiency) to identify which patients benefit the most from DCBA. Although SCBA have been widely used in the treatment of coronary artery disease, but the application in PAD has remained in the clinical trial phase. Most current clinical trials report only short-term (3–12 months) outcomes (41–43). Therefore, a future network meta-analysis could be conducted to compare the long-term real-world outcomes of SCBA, PCBA, and POBA in the treatment of PAD. Moreover, there is a need for further studies on the long-term safety of DCBA, particularly regarding the potential systemic toxicity of paclitaxel, including its effects on long-term cardiovascular events and survival rates. As healthcare costs continue to rise, cost-effectiveness analyses of DCBA in different health economic settings will provide valuable insights for clinical decision-making and policy development. Future exploration may also focus on the development of novel drug-coated balloons, optimizing drug delivery systems to enhance the stability and durability of the drug at the lesion site. Additionally, advanced imaging technologies could be employed to monitor treatment outcomes, further clarifying the long-term benefits of DCBA in improving patient prognosis and quality of life, thereby providing more robust evidence for the precise treatment of PAD.

## Data Availability

The original contributions presented in the study are included in the article/Supplementary Material, further inquiries can be directed to the corresponding author.
