# Supplementary material for: Comparison of clinical outcomes of drug-coated balloons angioplasty vs. plain old balloons angioplasty for peripheral arterial disease: an umbrella meta-analysis
Source: Front Cardiovasc Med. 2024 Nov 21;11:1511268. doi: 10.3389/fcvm.2024.1511268 (PMC11617568; doi:10.3389/fcvm.2024.1511268)
Supplement: Supplementary file 1 [file Supplementaryfile1.docx]

**Supplementary**

**Supplementary Table 1. PRISMA Checklist**

| **Section and Topic** | **Item #** | **Checklist item** | **Location where item is reported** |
| --- | --- | --- | --- |
| **TITLE** | | |  |
| Title | 1 | Identify the report as a systematic review. | Title page |
| **ABSTRACT** | | |  |
| Abstract | 2 | See the PRISMA 2020 for Abstracts checklist. | Abstract |
| **INTRODUCTION** | | |  |
| Rationale | 3 | Describe the rationale for the review in the context of existing knowledge. | BACKGROUND: 1^st^ paragraph to 4^th^ paragraph |
| Objectives | 4 | Provide an explicit statement of the objective(s) or question(s) the review addresses. | BACKGROUND: 5^th^ paragraph |
| **METHODS** | | |  |
| Eligibility criteria | 5 | Specify the inclusion and exclusion criteria for the review and how studies were grouped for the syntheses. | MATERIALS AND METHODS: Inclusion and Exclusion Criteria |
| Information sources | 6 | Specify all databases, registers, websites, organisations, reference lists and other sources searched or consulted to identify studies. Specify the date when each source was last searched or consulted. | MATERIALS AND METHODS: Study Registration, Search Strategy |
| Search strategy | 7 | Present the full search strategies for all databases, registers and websites, including any filters and limits used. | MATERIALS AND METHODS: Search Strategy, Supplementary Table 2 |
| Selection process | 8 | Specify the methods used to decide whether a study met the inclusion criteria of the review, including how many reviewers screened each record and each report retrieved, whether they worked independently, and if applicable, details of automation tools used in the process. | MATERIALS AND METHODS: Inclusion and Exclusion Criteria |
| Data collection process | 9 | Specify the methods used to collect data from reports, including how many reviewers collected data from each report, whether they worked independently, any processes for obtaining or confirming data from study investigators, and if applicable, details of automation tools used in the process. | MATERIALS AND METHODS: Data Extraction |
| Data items | 10a | List and define all outcomes for which data were sought. Specify whether all results that were compatible with each outcome domain in each study were sought (e.g. for all measures, time points, analyses), and if not, the methods used to decide which results to collect. | MATERIALS AND METHODS: Data Extraction, Supplementary Table 3 |
|  | 10b | List and define all other variables for which data were sought (e.g. participant and intervention characteristics, funding sources). Describe any assumptions made about any missing or unclear information. | MATERIALS AND METHODS: Data Extraction, Supplementary Table 3 |
| Study risk of bias assessment | 11 | Specify the methods used to assess risk of bias in the included studies, including details of the tool(s) used, how many reviewers assessed each study and whether they worked independently, and if applicable, details of automation tools used in the process. | MATERIALS AND METHODS: Quality Assessment, Supplementary Table 4 |
| Effect measures | 12 | Specify for each outcome the effect measure(s) (e.g. risk ratio, mean difference) used in the synthesis or presentation of results. | MATERIALS AND METHODS: Data Analysis |
| Synthesis methods | 13a | Describe the processes used to decide which studies were eligible for each synthesis (e.g. tabulating the study intervention characteristics and comparing against the planned groups for each synthesis (item #5)). | MATERIALS AND METHODS: Data Analysis |
|  | 13b | Describe any methods required to prepare the data for presentation or synthesis, such as handling of missing summary statistics, or data conversions. | MATERIALS AND METHODS: Data Analysis |
|  | 13c | Describe any methods used to tabulate or visually display results of individual studies and syntheses. | MATERIALS AND METHODS: Data Analysis |
|  | 13d | Describe any methods used to synthesize results and provide a rationale for the choice(s). If meta-analysis was performed, describe the model(s), method(s) to identify the presence and extent of statistical heterogeneity, and software package(s) used. | MATERIALS AND METHODS: Data Analysis |
|  | 13e | Describe any methods used to explore possible causes of heterogeneity among study results (e.g. subgroup analysis, meta-regression). | MATERIALS AND METHODS: Data Analysis |
|  | 13f | Describe any sensitivity analyses conducted to assess robustness of the synthesized results. | MATERIALS AND METHODS: Data Analysis |
| Reporting bias assessment | 14 | Describe any methods used to assess risk of bias due to missing results in a synthesis (arising from reporting biases). | MATERIALS AND METHODS: Quality Assessment |
| Certainty assessment | 15 | Describe any methods used to assess certainty (or confidence) in the body of evidence for an outcome. | MATERIALS AND METHODS: Data Analysis |
| **RESULTS** | | |  |
| Study selection | 16a | Describe the results of the search and selection process, from the number of records identified in the search to the number of studies included in the review, ideally using a flow diagram. | RESULTS: Study Characteristics, Figure 1 |
|  | 16b | Cite studies that might appear to meet the inclusion criteria, but which were excluded, and explain why they were excluded. | Figure 1 |
| Study characteristics | 17 | Cite each included study and present its characteristics. | Table 1 |
| Risk of bias in studies | 18 | Present assessments of risk of bias for each included study. | Table 1, Supplementary Table 4 |
| Results of individual studies | 19 | For all outcomes, present, for each study: (a) summary statistics for each group (where appropriate) and (b) an effect estimate and its precision (e.g. confidence/credible interval), ideally using structured tables or plots. | Table 1 |
| Results of syntheses | 20a | For each synthesis, briefly summarise the characteristics and risk of bias among contributing studies. | RESULTS: Publication Bias, Figure 2, Figure 3 |
|  | 20b | Present results of all statistical syntheses conducted. If meta-analysis was done, present for each the summary estimate and its precision (e.g. confidence/credible interval) and measures of statistical heterogeneity. If comparing groups, describe the direction of the effect. | RESULTS: Main Analysis of Clinical outcomes, Figure 2, Figure 3 |
|  | 20c | Present results of all investigations of possible causes of heterogeneity among study results. | RESULTS: Subgroup Analysis of Primary Outcomes, Table 2 |
|  | 20d | Present results of all sensitivity analyses conducted to assess the robustness of the synthesized results. | RESULTS: Sensitivity Analysis, Supplementary Figure 1, Supplementary Figure 2 |
| Reporting biases | 21 | Present assessments of risk of bias due to missing results (arising from reporting biases) for each synthesis assessed. | RESULTS: Publication Bias, Supplementary Figure 3 |
| Certainty of evidence | 22 | Present assessments of certainty (or confidence) in the body of evidence for each outcome assessed. | RESULTS: Main Analysis of Clinical outcomes, Figure 2, Figure 3 |
| **DISCUSSION** | | |  |
| Discussion | 23a | Provide a general interpretation of the results in the context of other evidence. | DISCUSSION: 1^st^ paragraph to 4^th^ paragraph |
|  | 23b | Discuss any limitations of the evidence included in the review. | DISCUSSION: 5^th^ paragraph |
|  | 23c | Discuss any limitations of the review processes used. | DISCUSSION: 5^th^ paragraph |
|  | 23d | Discuss implications of the results for practice, policy, and future research. | CONCLUSIONS |
| **OTHER INFORMATION** | | |  |
| Registration and protocol | 24a | Provide registration information for the review, including register name and registration number, or state that the review was not registered. | MATERIALS AND METHODS: Study Registration |
|  | 24b | Indicate where the review protocol can be accessed, or state that a protocol was not prepared. | MATERIALS AND METHODS: Study Registration |
|  | 24c | Describe and explain any amendments to information provided at registration or in the protocol. | Not applicable |
| Support | 25 | Describe sources of financial or non-financial support for the review, and the role of the funders or sponsors in the review. | FUNDING |
| Competing interests | 26 | Declare any competing interests of review authors. | CONFLICT OF INTEREST |
| Availability of data, code and other materials | 27 | Report which of the following are publicly available and where they can be found: template data collection forms; data extracted from included studies; data used for all analyses; analytic code; any other materials used in the review. | DATA AVAILABILITY STATEMENT |

**Supplementary Table 2. Search Strategy and Results**

| Database or registers | Full search strategy for each databases | Citations |
| --- | --- | --- |
| Cochran Library | #1 ("drug coated balloon"):ti,ab,kw OR (DCB):ti,ab,kw OR ("drug-coated balloon"):ti,ab,kw OR (angioplasty):ti,ab,kw OR ("drug coated balloon angioplasty"):ti,ab,kw OR (drug-coated):ti,ab,kw  #2 ("peripheral arterial disease"):ti,ab,kw OR ("critical limb ischemia"):ti,ab,kw OR ("arterial occlusive disease"):ti,ab,kw OR ("arterial occlusive disease"):ti,ab,kw (Word variations have been searched)  #3 ("meta analysis"):ti,ab,kw OR (""meta analyses""):ti,ab,kw  #1 AND #2 AND #3 (with limits: Date published on the Cochrane Library between January, 2014 and September, 2024) | 26 |
| Embase | #1 Title, abstract, keywords: “drug coated balloon” or “DCB” or “angioplasty” or “drug coated balloon angioplasty” or “drug-coated*” or “drug-coated balloon”  #2 Title, abstract, keywords: “peripheral arterial disease” or “critical limb isch*” or “arterial occlusive disease” or “arterial occlusive disease”  #3 Title, abstract, keywords: “meta analy*”  #4 Year(s): 2014-2024  #5 #1 AND #2 AND #3 AND #4 | 68 |
| PubMed | ("meta analy*"[Title/Abstract] AND ("peripheral arterial disease"[Title/Abstract] OR "critical limb isch*"[Title/Abstract] OR "arterial occlusive disease"[Title/Abstract] OR "arterial occlusive disease"[Title/Abstract]) AND ("drug coated balloon"[Title/Abstract] OR "DCB"[Title/Abstract] OR "angioplasty"[Title/Abstract] OR "drug coated balloon angioplasty"[Title/Abstract] OR "drug coated*"[Title/Abstract] OR "drug coated balloon"[Title/Abstract])) AND (2014:2024[pdat]) | 104 |
| Web of Science | 1# "drug coated balloon" (Topic) or DCB (Topic) or “angioplasty” (Topic) or “drug coated balloon angioplasty” (Topic) or “drug-coated*” (Topic) or “drug-coated balloon” (Topic)  2# “peripheral arterial disease” (Topic) or “critical limb isch*” (Topic) or “arterial occlusive disease” (Topic) or “arterial occlusive disease” (Topic)  3# "meta analy*" (Topic)  4# 2014-2024 (Year Published)  5 # 1# AND #2 AND #3 AND #4 | 289 |

**Supplementary Table 3. Definitions of Outcomes**

| Category | Outcome | Definition |
| --- | --- | --- |
| Primary outcomes | TLR | Any clinically driven repeat percutaneous intervention of the target lesion or bypass surgery of the target vessel. |
|  | PP | Absence of recurrent target lesion stenosis > 50% by imaging that is obtained without the need for additional or secondary surgical or endovascular procedures. |
|  | ACM | Death due to any specific cause, direct or indirect. |
|  | Amputation | Major amputation above the ankle. |
| Secondary outcomes | MAE | Composite of all-cause mortality, major amputation and TLR. |
|  | Restenosis | >50% recurrent stenosis on duplex ultrasound or angiography, or a peak systolic velocity rate ≥2.5 on duplex ultrasound. |
|  | LLL | Change in minimum lumen diameter from the final angiogram to follow-up. |
|  | ABI | Ratio of ankle artery pressure to brachial artery pressure. |

**Supplementary Table 4. Results of AMSTAR 2 Assessment**

| Item*  Author, year | 1 | 2 | 3 | 4 | 5 | 6 | 7 | 8 | 9 | 10 | 11 | 12 | 13 | 14 | 15 | 16 | Total score |
| --- | --- | --- | --- | --- | --- | --- | --- | --- | --- | --- | --- | --- | --- | --- | --- | --- | --- |
| Katsanos, 2016 | yes | yes | yes | partial yes | yes | yes | partial yes | partial yes | yes | no | yes | yes | yes | yes | yes | yes | 15 |
| Cassese, 2016 | no | yes | yes | partial yes | yes | no | partial yes | partial yes | yes | no | yes | no | no | no | yes | no | 9 |
| Caradu, 2019 | no | partial yes | yes | partial yes | no | no | partial yes | yes | yes | no | yes | yes | yes | yes | yes | yes | 12 |
| Klumb, 2019 | yes | partial yes | yes | partial yes | yes | yes | partial yes | yes | yes | no | yes | no | yes | yes | yes | no | 13 |
| Varetto, 2019 | yes | partial yes | yes | partial yes | no | no | partial yes | partial yes | yes | no | yes | no | yes | yes | yes | yes | 12 |
| Anantha-Narayanan, 2019 | yes | partial yes | yes | partial yes | yes | yes | no | partial yes | yes | no | yes | no | yes | yes | yes | yes | 13 |
| Ipema, 2020 | yes | partial yes | yes | partial yes | yes | no | partial yes | yes | yes | no | yes | no | yes | no | no | yes | 11 |
| Dinh, 2021 | yes | partial yes | yes | partial yes | yes | yes | yes | yes | yes | no | yes | no | yes | yes | yes | yes | 14 |
| Cao, 2021 | yes | partial yes | yes | partial yes | yes | yes | yes | yes | yes | no | yes | no | yes | yes | yes | yes | 14 |
| Zhang, 2022 | yes | yes | yes | partial yes | yes | yes | partial yes | partial yes | yes | no | yes | no | yes | yes | yes | no | 13 |
| Cai, 2022 | yes | partial yes | yes | partial yes | yes | yes | yes | partial yes | yes | no | yes | yes | yes | yes | yes | no | 14 |
| Barbarawi, 2022 | yes | partial yes | yes | partial yes | yes | yes | no | partial yes | yes | no | yes | no | yes | no | yes | no | 11 |
| Ullah, 2022 | yes | partial yes | yes | no | no | no | no | no | yes | no | yes | no | no | yes | yes | yes | 8 |
| Zhen, 2022 | yes | partial yes | yes | partial yes | yes | no | no | partial yes | yes | no | yes | no | no | yes | no | yes | 10 |
| Koeckerling, 2023 | yes | yes | yes | partial yes | yes | no | partial yes | partial yes | yes | no | yes | no | yes | yes | yes | yes | 13 |
| Cui, 2024 | yes | partial yes | yes | partial yes | no | yes | partial yes | yes | yes | no | yes | yes | yes | no | yes | no | 12 |

*Specific items:

1. Did the research questions and inclusion criteria for the review include the components of PICO?
2. Did the report of the review contain an explicit statement that the review methods were established prior to the conduct of the review and did the report justify any significant deviations from the protocol?
3. Did the review authors explain their selection of the study designs for inclusion in the review?
4. Did the review authors use a comprehensive literature search strategy?
5. Did the review authors perform study selection in duplicate?
6. Did the review authors perform data extraction in duplicate?
7. Did the review authors provide a list of excluded studies and justify the exclusions?
8. Did the review authors describe the included studies in adequate detail?
9. Did the review authors use a satisfactory technique for assessing the risk of bias (RoB) in individual studies that were included in the review?
10. Did the review authors report on the sources of funding for the studies included in the review?
11. If meta-analysis was performed did the review authors use appropriate methods for statistical combination of results?
12. If meta-analysis was performed, did the review authors assess the potential impact of RoB in individual studies on the results of the meta-analysis or other evidence synthesis?
13. Did the review authors account for RoB in individual studies when interpreting/discussing the results of the review?
14. Did the review authors provide a satisfactory explanation for, and discussion of, any heterogeneity observed in the results of the review?
15. If they performed quantitative synthesis did the review authors carry out an adequate investigation of publication bias (small study bias) and discuss its likely impact on the results of the review?
16. Did the review authors report any potential sources of conflict of interest, including any funding they received for conducting the review?

**Supplementary Table 5. Main Analysis Results of Outcomes**

| Outcomes | Number of included meta-analyses | *I*^2^ (%) | *P* | Pooled effect size (95% *CI*) |
| --- | --- | --- | --- | --- |
| Primary outcomes |  |  |  |  |
| TLR | 13 | 64.3 | 0.008 | 0.41 (0.34, 0.49) |
| PP | 6 | 82.5 | <0.001 | 2.05 (1.53, 2.75) |
| ACM | 13 | 0.0 | 0.614 | 1.08 (1.00, 1.15) |
| Amputation | 10 | 19.9 | 0.260 | 1.08 (0.86, 1.36) |
| Secondary outcomes |  |  |  |  |
| MAE | 3 | 0.0 | 0.578 | 0.76 (0.59, 0.98) |
| Restenosis | 9 | 0.0 | 0.637 | 0.46 (0.41, 0.51) |
| LLL | 4 | 0.0 | 0.619 | -0.87 (-1.00, -0.74) |
| ABI | 3 | 0.0 | 0.731 | 0.01 (-0.03, 0.05) |

**
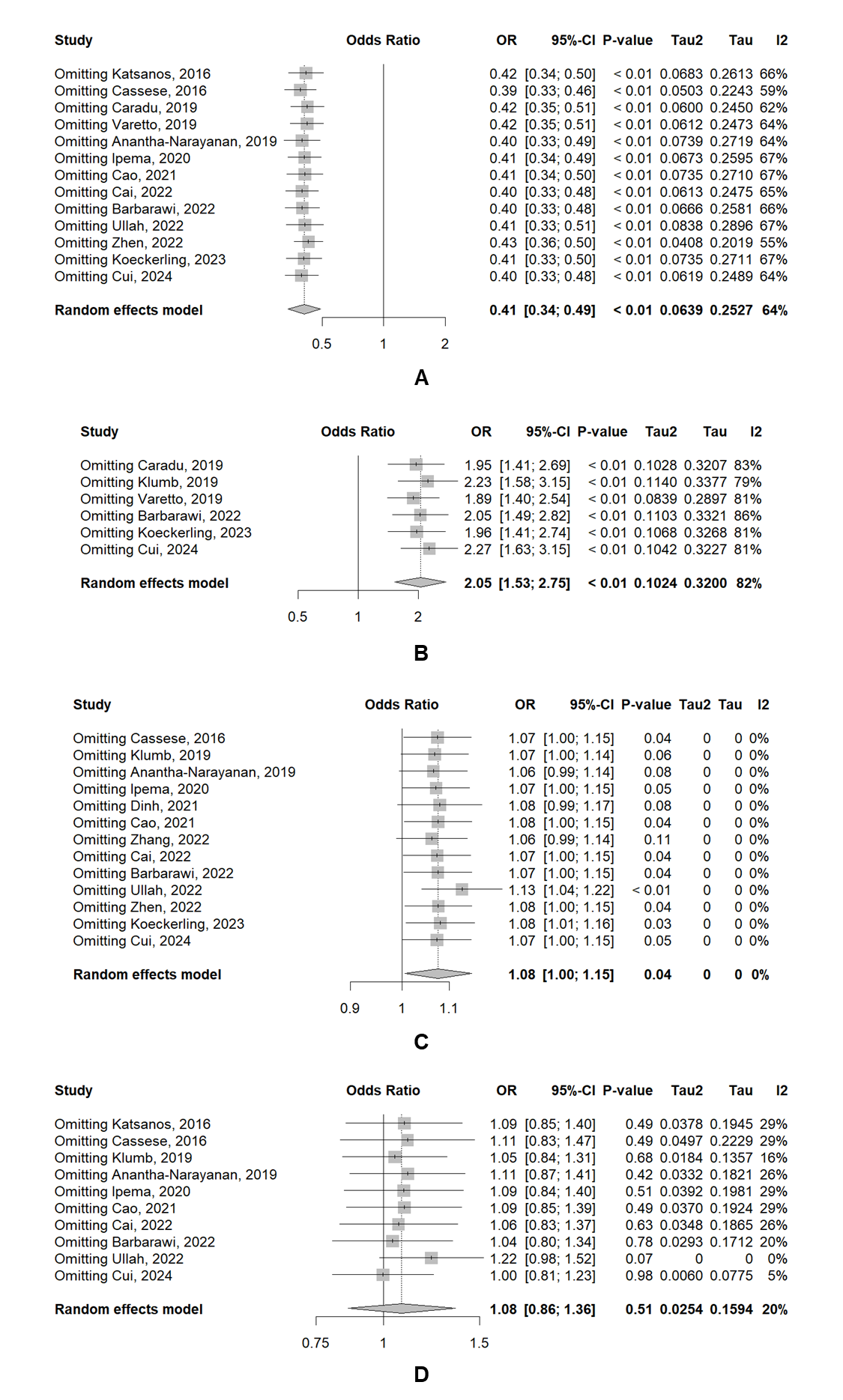
**

**Supplementary Figure 1. Sensitivity Analyses of Primary Outcome (A. TLR; B. PP; C: AMC; D. Amputation)**

**
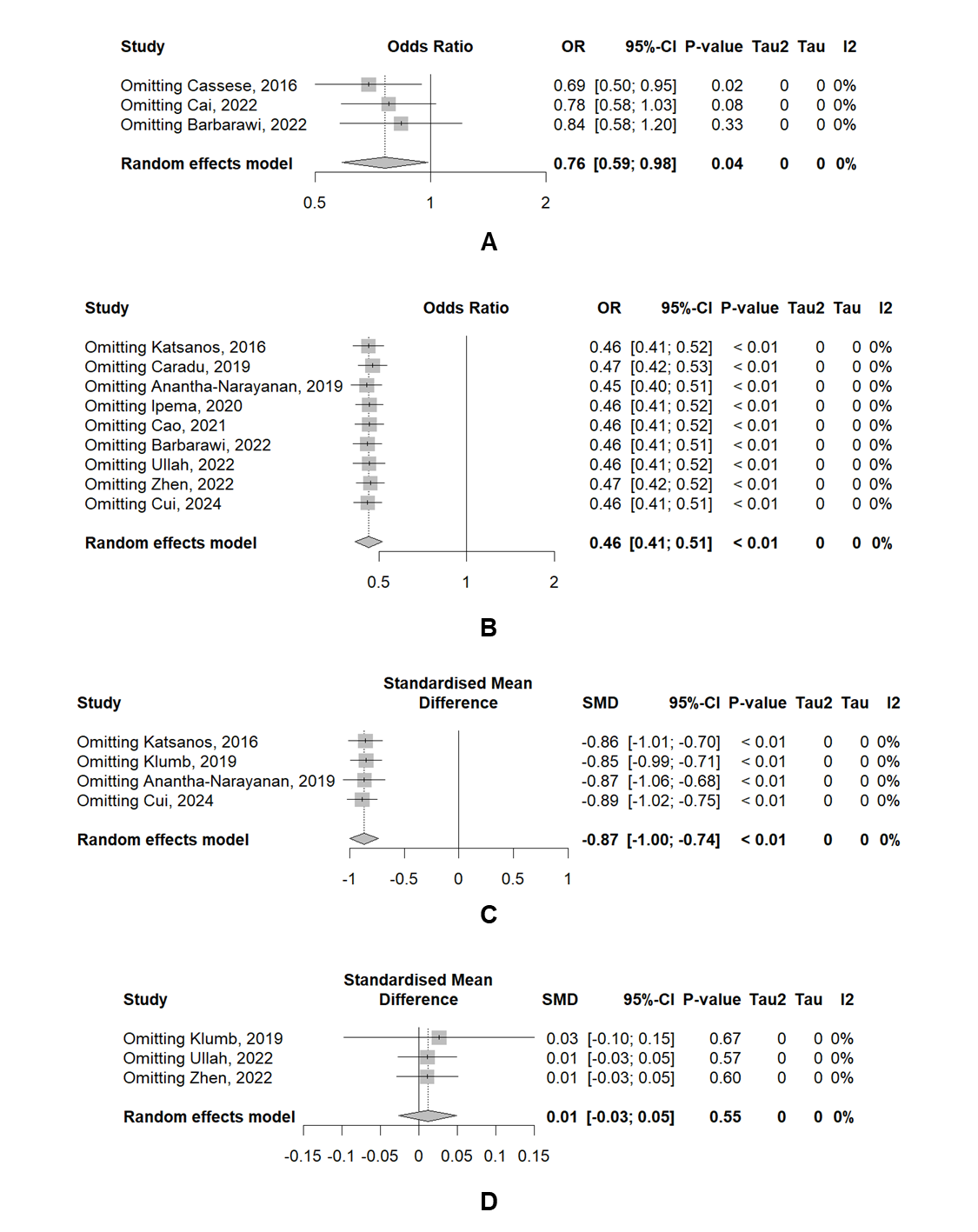
**

**Supplementary Figure 2. Sensitivity Analyses of Secondary Outcome(A. TLR; B. PP; C: AMC; D. Amputation)**

**
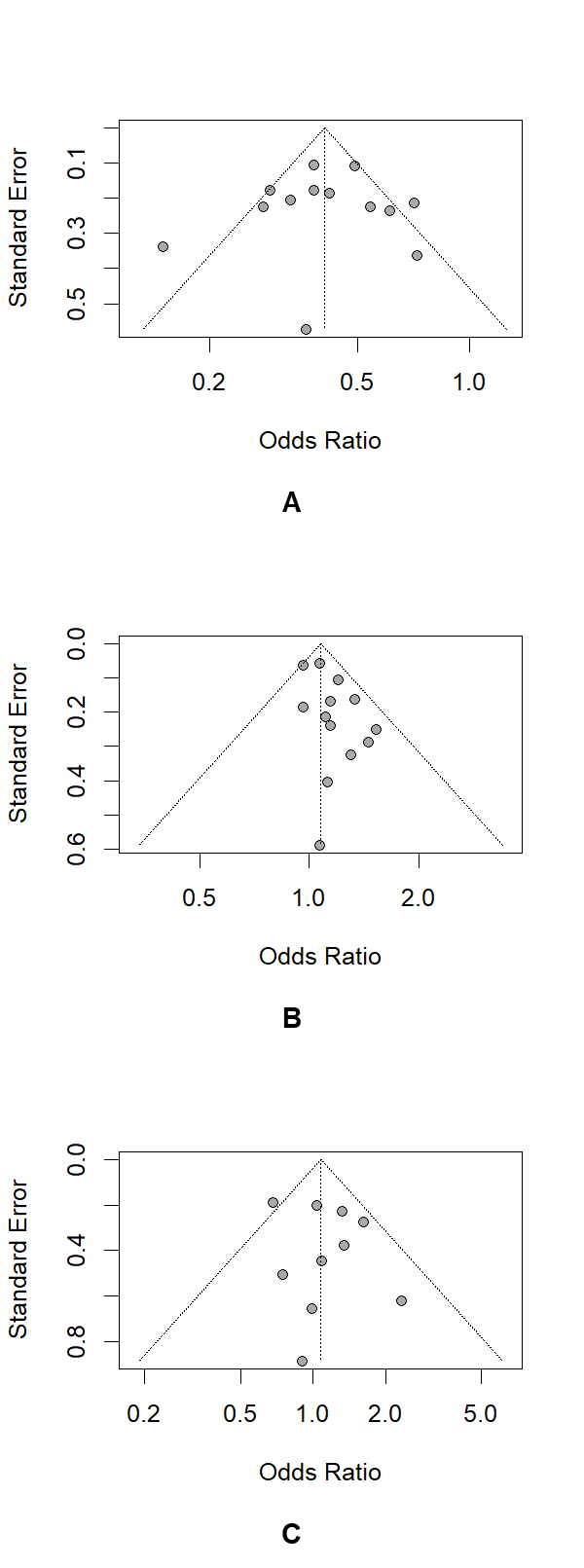
**

**Supplementary Figure 3. Funnel Plots of Meta-analyses of TLR, ACM, and Amputation (A. TLR; B. AMC; C: Amputation)**
